# PDGF/PDGFR: A Possible Molecular Target in Scleroderma Fibrosis

**DOI:** 10.3390/ijms23073904

**Published:** 2022-03-31

**Authors:** Chiara Paolini, Silvia Agarbati, Devis Benfaremo, Matteo Mozzicafreddo, Silvia Svegliati, Gianluca Moroncini

**Affiliations:** 1 Department of Clinical and Molecular Sciences, Università Politecnica delle Marche, 60126 Ancona, Italy; c.paolini@staff.univpm.it (C.P.); s.agarbati@staff.univpm.it (S.A.); d.benfaremo@staff.univpm.it (D.B.); m.mozzicafreddo@staff.univpm.it (M.M.); s.svegliati@staff.univpm.it (S.S.); 2 Department of Internal Medicine, Clinica Medica, Ospedali Riuniti “Umberto I-G.M. Lancisi-G. Salesi”, 60126 Ancona, Italy

**Keywords:** platelet-derived growth factor (PDGF), PDGF receptor (PDGFR), fibrosis, systemic sclerosis (SSc), scleroderma (SSc), anti-PDGFR autoantibodies, PDGF/PDGFR targeting therapy

## Abstract

Systemic sclerosis (SSc) is a clinically heterogeneous disorder of the connective tissue characterized by vascular alterations, immune/inflammatory manifestations, and organ fibrosis. SSc pathogenesis is complex and still poorly understood. Therefore, effective therapies are lacking and remain nonspecific and limited to disease symptoms. In the last few years, many molecular and cellular mediators of SSc fibrosis have been described, providing new potential options for targeted therapies. In this review: (i) we focused on the PDGF/PDGFR pathway as key signaling molecules in the development of tissue fibrosis; (ii) we highlighted the possible role of stimulatory anti-PDGFRα autoantibodies in the pathogenesis of SSc; (iii) we reported the most promising PDGF/PDGFR targeting therapies.

## 1. Introduction

Systemic sclerosis (SSc) is a rare, immune-mediated, connective tissue disease characterized by progressive fibrosis of the skin and internal organs. The hallmarks of this disease are microvascular lesions, the production of autoantibodies, and increased deposition of extracellular matrix (ECM).

SSc predominantly affects the female sex and its progression and severity vary greatly amongst individuals. The variable extent of fibrosis of the skin, lungs, and gastrointestinal (GI) tract is the most frequent manifestation of the disease, with the leading causes of death being interstitial lung disease (ILD) and pulmonary hypertension (PAH) [1,2].

The pathogenesis of this complex systemic disease is not completely understood. The heterogeneity of SSc clinical manifestations likely reflects the interplay of multiple pathogenic pathways, some of which are well characterized but not clearly associated with disease subsets and clinical phenotypes [3,4]. Much recent progress has been made with the contribution of new experimental models and omics approaches [5,6,7,8]. Nevertheless, there are no specific targeted therapies approved, with the exception of ERAs [9,10] and nintedanib [11]. Optimal clinical management of SSc is, therefore, hampered by a lack of drugs.

To possibly fill this gap, this article reviews the role of PDGF and its receptor as a key signaling pro-fibrotic pathway and potential therapeutic target in SSc.

## 2. Platelet-Derived Growth Factor (PDGF)

Platelet-derived growth factor (PDGF) was identified more than three decades ago as a serum growth factor for fibroblasts, smooth muscle cells (SMCs), and glial cells [12]. Moreover, it is the major mitogen for connective tissue cells and certain other cell types [13]. PDGF stimulates cell survival, growth, and proliferation, but also changes cell shape and motility; it induces a reorganization of the actin filament system and stimulates chemotaxis, i.e., a directed cell movement toward a gradient of PDGF [14].

Four distinct PDGF polypeptide chains have been described, namely, the PDGF-A, PDGF-B, PDGF-C, and PDGF-D, which form four homodimers including PDGF-AA, PDGF-BB, PDGF-CC, and PDGF-DD, as well as one heterodimer, PDGF-AB.

Different PDGF isoforms are generally produced by discrete populations of cells (i.e., platelets, endothelial, epithelial, glial, and inflammatory cells) [15], and act locally to drive different cellular responses [12].

PDGF expression in cultured cells is dynamic and responsive to a variety of stimuli, including hypoxia, thrombin, cytokines, and growth factors, including PDGF itself [12,16]. PDGF has an important role during embryonic development, functioning at the sites of epithelial–mesenchymal interactions during organogenesis, as well as during wound healing [13]. It also controls cell differentiation and stem cell pluripotency, along with mesenchymal stem cell (MSC) stroma formation and differentiation through negative regulation [17].

However, PDGF deregulation has been associated with several pathological conditions, such as atherosclerosis, organ fibrosis, and tumorigenesis [18,19].

## 3. PDGF Receptor (PDGFR)

The different isoforms of PDGF exert their biological effects on target cells by binding and activating PDGFRα and PDGFRβ, two structurally related class III tyrosine kinase receptors (RTKs) with different expression patterns and physiological roles. PDGFRs are transmembrane glycoproteins that include three kinds of dimers, αα, αβ, and ββ, which are composed of PDGF-α and PDGFR-β subunits in a certain way. PDGF-A and PDGF-C predominantly bind to the PDGFR-α chain, PDGF-B binds to both -α and -β chains, and PDGF-D binds to the β chain only.

PDGFR protein consists of an extracellular ligand-binding domain, a single transmembrane helix to pass structural/informational input from outside the cell, and an intracellular tyrosine kinase domain for the downstream transduction [20]. Downstream signal transduction pathways include phosphatidylinositol 2 kinase, MAPK, PI3K, Src family kinases, and phospholipase Cγ [21,22].

Compared to other RTKs, PDGFRs are featured by five immunoglobulin-like (Ig-like) domains in the extracellular segment (D1–D5), and a split kinases domain that contains an insert of variable length between the N- and C-terminals [23].

Two PDGFR genes *(PDGFRA* and *PDGFRB*), located on chromosomes 4 and 5 in humans and 5 and 18 in mice, respectively, are present [15].

PDGF receptors are tightly regulated by an auto-inhibitory allosteric conformation, resulting in very low basal activity in the absence of the ligand [24].

The best-known mechanism to activate PDGFRs is the PDGF-mediated (direct) activation mode, in which activated PDGFR tyrosine phosphorylates substrates engage signaling cascades that drive subsequent cellular responses [15]. For instance, both PDGFRα and PDGFRβ autophosphorylate and thereby create a binding site for SH2 domain-containing proteins such as phosphoinositide 3 kinase (PI3K). The relocation of PI3K to the plasma membrane increases its access to its lipid substrates and to co-activators such as activated Ras.

In different pathological conditions, PDGF-independent modes to activate PDGFRs were observed. For instance, growth factors outside of the PDGF family (non-PDGFs) were able to induce the PDGFRα indirect activation [25]. These non-PDGFs engage their own receptors and thereby trigger an intracellular signaling cascade that drives monomeric PDGFRα activation. It was discovered in the context of experimental PVR, where the pathogenesis depends on the prolonged activation of PDGFRα. VEGF enables indirect activation of PDGFRα by antagonizing PDGF-dependent activation of the receptor; removing VEGF allows PDGF to block the non-PDGFs, indirectly inhibiting activation of PDGFRα and thereby enabling the survival of cells displaced into the vitreous.

Several factors induce PDGFR expression, including TGF-β, estrogen, interleukin-1α (IL-1α), basic fibroblast growth factor-2 (FGF-2), tumor necrosis factor-α, and lipopolysaccharide.

PDGFRs are primarily expressed on mesenchymal cells. PDGFR activation exerts mitogenic and chemoattractant effects, which determine the main biological roles of PDGF in development, physiological, and pathological processes, e.g., cardiovascular and renal development, wound healing, atherosclerosis, malignancies, or fibrosis [26,27,28].

## 4. PDGF/PDGFR in Physiology and in Organ Fibrosis

Fibrosis is a pathological response to tissue wound healing that results in excessive formation and deposition of extracellular matrix (ECM) with significant architectural remodeling. It may occur in any organs and tissues; it may become irreversible with time and cause loss of organ function [17].

Multiple pathways are involved in the initiation and progression of organ fibrosis, with PDGF-signaling being one of the central mediators [29]. In fact, aberrant PDGFR signaling drives pathological responses in the principal cellular executors of fibrosis, i.e., mesenchymal stromal cells such as fibroblasts, pericytes, and myofibroblasts, in diverse fibrotic diseases [12], including cardiac and pulmonary fibrosis, liver cirrhosis, glomerulosclerosis, and systemic sclerosis [30,31,32].

PDGFs and their receptors contribute to normal heart development. Overexpression of human PDGF-A and PDGF-B genes in transgenic mice has a negative impact on cardiac development and function, resulting in isoform-specific fibrotic reactions and cardiac hypertrophy [33].

PDGFRα signaling promotes muscle development in growing embryos and angiogenesis in regenerating adult skeletal muscle. However, both increased PDGF ligand abundance and enhanced PDGFRα pathway activity cause pathological fibrosis, as seen in aged and diseased muscles. In mice, PDGFRα signaling regulates a population of muscle-resident fibro/adipogenic progenitors (FAPs) that play a supportive role in muscle regeneration but may also cause fibrosis when aberrantly regulated [34]. During muscle regeneration, FAPs produce an intronic variant of PDGFRα, which can inhibit PDGF signaling and prevent FAPs activation. Increasing the expression of this isoform limits fibrosis in mice.

## 5. PDGF/PDGFR in Preclinical Models of Systemic Sclerosis

Several recent findings confirm previous evidence suggesting that the PDGF/PDGFR pathway plays an important role in the development and progression of fibrosis in systemic sclerosis, being implicated in the activation of SSc fibroblasts [35].

PDGFs and PDGFRs are upregulated in fibrotic dermal lesions of patients with scleroderma [36,37]. 

Moreover, elevated levels of PDGF-A and PDGF-B are found in bronchoalveolar lavage (BAL) fluid obtained from scleroderma patients [38].

Increased PDGFRα signaling in both embryo and adult mice leads to connective tissue hyperplasia and increased extracellular matrix deposition, resulting in progressive, chronic fibrosis in multiple organs [24]. Vice versa, RNA interference against PDGFRα mRNA inhibits in vitro transdifferentiation of SSc dermal fibroblasts into myofibroblasts [39].

MicroRNA miR-30b, which in turn suppresses PDGFRβ expression, is strongly down-regulated in serum and in the affected skin of SSc patients [40]. The blocking of PDGFRβ by miR-30b transfection in SSc dermal fibroblasts in vitro significantly reduces αSMA and Col1A2 gene expression, inhibiting collagen synthesis and myofibroblast differentiation. Conversely, PDGFRβ activation by intradermal injection of PDGF-BB in mice significantly aggravates bleomycin-induced dermal thickening, vascular alterations, and monocyte/macrophage infiltration, exacerbating skin injury and fibrosis [41].

## 6. Anti-PDGFRα Autoantibodies in Systemic Sclerosis

Human PDGFRα is a target of the autoimmune response in systemic sclerosis. Stimulatory anti-PDGFRα autoantibodies were detected in the whole IgG purified from the serum of SSc patients [42]. Unlike other autoantibodies previously described in the literature [43], these autoantibodies possess a biological activity and may contribute, as pathogenic factors, to tissue damage. In fact, upon binding to the receptor on the fibroblast cell surface, they can abnormally activate it, selectively inducing the Ha-Ras-ERK1/2 signaling pathway and reactive oxygen species (ROS) production and stimulating type I collagen gene expression. Triggering this intracellular loop by specific autoantibodies against PDGFRα might sustain the myofibroblast phenotype conversion and the persistent profibrotic response of SSc fibroblasts, suggesting a causal role in the pathogenesis of scleroderma [44]. There are no studies indicating the mechanisms responsible for the generation of anti-PDGFRα autoantibodies. One of the following may be postulated: (i) anti-PDGFRα autoantibodies are the result of the general break of tolerance towards self-antigens, which is a hallmark of SSc disease; (ii) more specifically, PDGFRα peptides recognized by autoantibodies may be the result of intracellular splicing processes caused by external triggers such as viral agents, e.g., CMV [45].

To confirm the original discovery, the immune repertoire of one scleroderma patient was directly investigated [46]. Briefly, memory B cell clones specifically producing antibodies against the human PDGFRα were isolated from peripheral blood mononuclear cells (PBMCs); messenger RNA was extracted, antibody-heavy and light chain genes were PCR amplified and sequenced, and then cloned and recombined in vitro to obtain three novel human monoclonal antibodies. The latter replicated the anti-PDGFRα autoantibodies natively generated by the scleroderma patient. Interestingly, the three monoclonals, differing only in the light chain, display distinct functional features, depending on their epitopes, i.e., the different binding sites on the human PDGFRα: the first one binds with very low affinity to a single linear amino acid sequence in the first PDGFRα extracellular domain, without any stimulatory effects on human fibroblasts in vitro; the second one binds a discontinuous epitope within the first and the second PDGFRα extracellular domains, and induces the generation of ROS but not of collagen; the third one binds with high affinity a discontinuous epitope within the second and third PDGFRα extracellular domains, largely overlapping with the PDGF binding site, and induces both ROS and collagen production [46,47].

**Figure 1:**
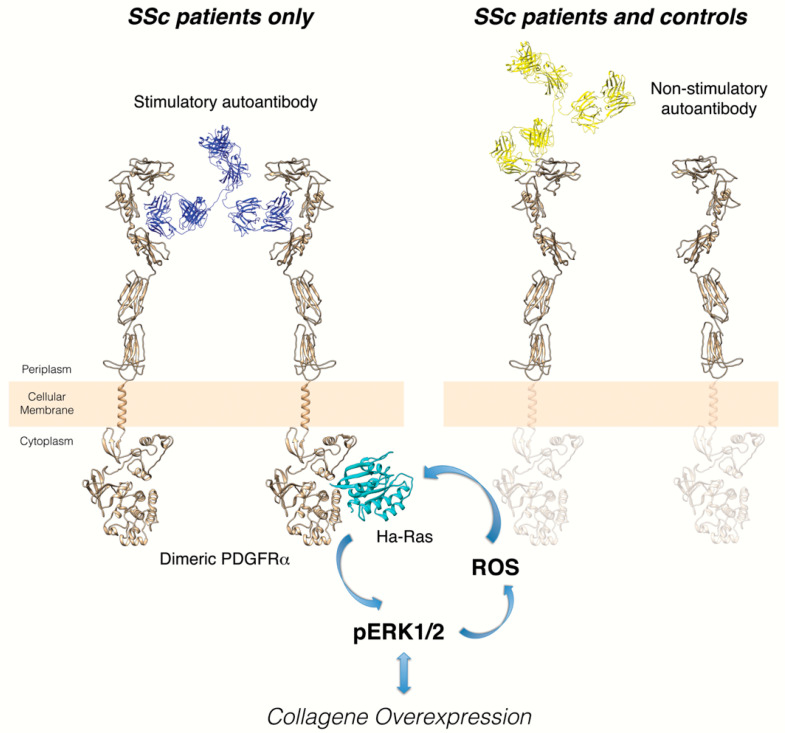
Distinct epitopes of stimulatory and non-stimulatory anti-PDGFRα autoantibodies. Left cartoon: the interaction of stimulatory autoantibody cloned from memory B cells of a scleroderma patient and the discontinuous, conformational epitope identified on the extracellular PDGFRα domain induces the intracellular signaling activation and collagen gene overexpression. The identification of this binding region of PDGFRα provides a possible target for new therapeutic strategies to block excessive collagen synthesis under pathologic conditions such as SSc. Right cartoon: binding of non-stimulatory autoantibodies cloned from memory B cells of the same scleroderma patient to a single linear epitope of extracellular PDGFRα domain does not induce receptor dimerization/activation. This non-agonistic autoantibody could reflect the natural autoimmunity, already observed in many healthy individuals, without any clear role in physiology or disease.

Through a large conformational PDGFRα peptide library, the epitope of the stimulatory anti-PDGFRα monoclonal autoantibody, overlapping with the PDGF binding site, was further characterized. In particular, the receptor regions required for binding were dissected from the minimal regions of the receptor mediating the functional activity of the stimulatory anti-PDGFRα monoclonal autoantibody. Moreover, based on these data and by using synthetic peptides mimicking this epitope, it was possible to detect antibodies with the same binding features in the serum of other SSc patients, but not in the serum of healthy or pathologic (Primary Raynaud’s Phenomenon, Systemic Lupus Erythematosus) individuals. On the contrary, non-stimulatory anti-PDGFRα autoantibodies were also found in a significant percentage of both control cohorts (Figure 1). These findings demonstrate that agonistic anti-PDGFRα autoantibodies directed toward stimulatory epitopes are SSc-specific [48].

This study is of particular interest for different reasons: (i) the existence of activating anti-PDGFRα autoantibodies in the memory B cells and serum of scleroderma patients has been confirmed, shedding light on the still-unclear pathogenesis of one of the most important autoimmune diseases; (ii) the study provides novel evidence about the involvement of PDGFRα in SSc pathogenesis, identifying potentially useful new therapeutic targets for this drug-orphan disease and other fibrotic disorders; (iii) the identification of selective immunodominant epitopes discriminating between SSc and control sera could contribute to developing novel diagnostic assays.

Stimulatory autoantibodies against human PDGFRα have also been demonstrated to activate human smooth muscle cells in vitro and may thus contribute to the development of SSc vascular lesions [49]. In fact, upon incubation with PDGF or with agonistic anti-PDGFRα autoantibodies, but not with normal IgG, human pulmonary artery smooth muscle cells (HPASMC) displayed a pro-fibrotic synthetic phenotype as expressed by high levels of type-I collagen and myofibroblastic marker, α-SMA and a higher growth rate and reduced expression of markers characteristic of the contractile phenotype such as smooth-muscle myosin heavy chain and smooth-muscle calponin. 

Furthermore, the profibrotic role of stimulatory anti-PDGFRα autoantibodies was demonstrated also in vivo by intradermal injection in three-dimensional bioengineered skin samples, containing human keratinocytes and fibroblasts isolated from skin biopsies of healthy donors, grafted onto the back of SCID mice [50]. An agonistic anti-PDGFRα monoclonal antibody, but not the non-agonistic one, induced enhanced collagen deposition, increased fibroblast activation markers, and vascular alterations in the dermis of human skin equivalents, leading to scleroderma-like skin fibrosis.

## 7. Structural Analysis of PDGFRα and its Ligands

The three-dimensional structure of the PDGFRα extracellular domain, complexed or not with ligands, has been inferred from in silico predicted analyses, since a complete crystallographic structure of this receptor is still missing. The only current available X-ray crystallographic structure (pdbID: 7lbf) of PDGFRα, although partial, was published by Kschonsak et al. in 2021 [51]. Here, the authors presented the molecular complex between PDGFRα and HCMV Trimer, involving four binding sites on the receptor: S1 and S2 placed into the D1, S3 into the D2, and S4 into the D3 domain.

However, a reliable and complete homology modelled structure of human PDGFRα extracellular domain is obtainable, as previously reported by us [46], using templates with relatively high structural and sequence similarities such as the crystal structures of human PDGFRβ complex (pdbID: 3mjg) [52], of the extracellular domain of human receptor tyrosine kinase, Kit (pdbID: 2e9w) [53], or of human vascular endothelial growth factor receptor 1, VEGFR-1 (pdbID: 5t89) [54]. 

Our study [46] confirmed the crystal structure of the PDGF/PDGFRβ complex by performing a molecular docking analysis, based on the rigid body protein–protein geometric approach between the homology modelled PDGFRα extracellular domain and the crystallographic structure of PDGF (pdbID: 1pdg) [55]. We found the same corresponding amino acids, included in the large cleft at the D2-D3 boundary for interaction with PDGF-B, as also described by Chen et al. [23]. The latter study reported that the replacement of these aromatic residues in PDFGRβ with non-aromatic residues in PDGFRα makes the ligand recognition surface of PDGFRα more adaptive to a wider variety of PDGF ligand surfaces. For example, the HCMV Trimer binds with high affinity and high selectivity to PDGFRα, but it does not bind to the closely related PDGFRβ.

**Figure 2:**
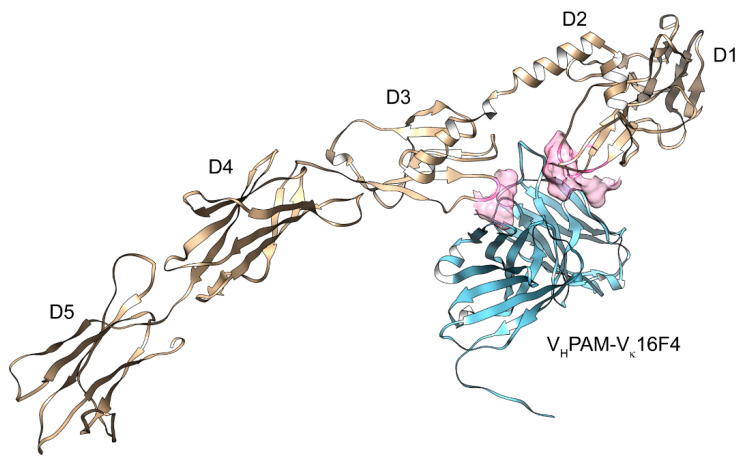
Molecular modeling of the V_H_PAM–V_κ_16F4 (light blue)/PDGFRα (gold) complex. The identification of the five immunoglobulin-like (Ig) domains in the extracellular segment (D1–D5) and the binding area (in pink surface) of the PDGF-B/PDGFRα complex are reported.

In this regard, in the same study [46], we also reported the predicted, subsequently validated binding of the aforementioned monoclonal autoantibodies with the PDGFRα. Some predicted models (in particular, for the V_H_PAM–V_κ_16F4/PDGFRα complex) showed binding regions superimposable with the conformational and discontinuous site related to the binding of PDGF/PDGFRα (Figure 2), further confirming the previous adaptive hypothesis suggested by Chen et al. Moreover, additional models showed two other different binding sites/epitopes—one placed into the D1–D2 boundary (for the V_H_PAM–V_λ_16F4/PDGFRα complex) and the other one located onto the D1 domain (for the V_H_PAM–V_κ_13B8/PDGFRα complex)—that would produce partial or no responses into the cell. All these models were then confirmed by screening a conformational PDGFRα peptide library with these ligands by binding competition experiments performed on a surface plasmon resonance device, and by analysis of ROS production and type I collagen gene induction in human fibroblasts.

In addition, the crystal structure of the globular PDGFRα kinase domain, free or bound to small ligands/drugs (WQ-C-159, crenolanib, sunitinib, and imatinib), was well characterized [56]. Its structure shows a typical type III RTK composition incorporating a bilobal kinase, an activation loop, and a JM domain, and it is very similar to the structure of auto-inhibited FLT3 and c-Kit.

## 8. PDGF/PDGFR Targeting Approaches in Systemic Sclerosis

PDGF is a well-described mediator of fibrosis, and its modulation is a promising option in an anti-fibrotic treatment [17].

In general, three main approaches can be conceived to inhibit the PDGF/PDGFR pathway: (1) sequestering the ligand with neutralizing antibodies, or soluble extracellular fragments of the receptors acting as ligand traps/decoy receptors or DNA/RNA aptamers; (2) disrupting the interaction between the receptor and the ligand by blocking the receptor with receptor-specific antibodies or small molecule inhibitors; (3) using low molecular weight inhibitors to block the kinase activity of the PDGFR [57].

Table 1 summarizes the possible pharmacological targeting of the PDGF/PDGFR pathway.

There are several studies focused on the efficacy of PDGF/PDGFR-blocking antibodies. 

Structural studies on the PDGF-B blocking antibody MOR8457 showed that it causes a conformational change of PDGF-B that prevents binding to PDGFRβ [58]. Moreover, Muse H. et al. discovered that a PDGF-AB neutralizing antibody prevented electromechanical remodeling of adult atrial myocytes in a co-culture model with a myofibroblast [59].

In addition to blocking the ligand, there are several strategies that block the receptor. 

PDGFR-specific neutralizing antibodies block signaling through either PDGFRα or PDGFRβ receptors, reducing PDGF-AA and PDGF-BB-induced collagen gel remodeling and migration in human dermal and lung fibroblasts in vitro [60].

Olaratumab is a recombinant human IgG1 anti-PDGFRα monoclonal antibody that binds specifically to PDGFRα, blocking PDGF-AA, PDGF-BB, and PDGF-CC binding and receptor activation. Through this mechanism, it reduces the proliferation of several tumor models both in vitro and in vivo [61,62]. Olaratumab was initially evaluated and preliminarily approved for the treatment of patients with advanced soft-tissue sarcomas but, following the disappointing results of the phase III study, approval for clinical use was withdrawn [63].

To date, the most effective approach to decreasing PDGFR signaling is to block its enzymatic activity; the inhibition of kinase activity of PDGFRs is based on multi-targeted small molecules, called tyrosine kinase inhibitors (TKI). TKIs are not selective for the PDGFR but their mechanism of action encompasses several other kinases such as, for example, c-KIT and Abl for imatinib or FGFR and VEGFR for nintedanib [63].

Imatinib was the first TKI developed and approved for clinical use in hematologic cancers [64]. Current indications for imatinib include hematologic neoplasms (such as chronic myeloid leukemia and acute lymphoblastic leukemia) and gastrointestinal stromal tumors (GIST). Imatinib is a potent inhibitor of TK phosphorylation that prevents the binding of ATP to the target kinase. Imatinib inhibits collagen and ECM production induced by PDGF, but also, through a non-canonical pathway, by TFG-β [65].

Several studies tested the potential use of imatinib as an antifibrotic drug for the treatment of dermal fibrosis in systemic sclerosis. In preliminary studies, treatment with imatinib reduced dermal thickness and prevented the differentiation of fibroblasts into myofibroblasts, both in vitro and in vivo [66,67]. 

Following the preclinical results, several clinical studies evaluated imatinib use in patients with systemic sclerosis [68]. Although encouraging results were reported by several uncontrolled studies, clinical outcomes were highly variable and often limited by important toxicity. Subsequent randomized and controlled trials yielded mostly negative results, reporting that imatinib use was not associated with improvement of cutaneous involvement, although it may determine the stabilization of lung function [65,69].

As recently observed by Harrach et al. [70], the reason for insufficient clinical responses to imatinib therapy in SSc patients may be partly attributable to the reduced drug transporter expression, lowering the intracellular imatinib concentrations and thus the therapeutic outcome. In fact, PDGF significantly reduced imatinib influx into SSc fibroblasts by decreasing gene transcription and subsequent protein expression of the transporter MATE1, whereas the transport process in healthy fibroblasts remained unaffected. Because MATE1 expression is regulated by Notch signaling, the blockade of the deregulated Notch signaling network normalized the transporter function of MATE1, presumably rescuing the decreased imatinib efficacy in SSc fibroblasts.

Crenolanib besylate is a selective inhibitor of type III tyrosine kinases with higher sensitivity for PDGFRα than PDGFRβ [71]. It can block, in a dose-dependent manner, PDGFRα and PDGFRβ phosphorylation upon stimulation with PDGF-AA and/or PDGF-BB in SSc and healthy control dermal fibroblast and can inhibit their proliferation and migration in vitro. A recent study demonstrated that crenolanib abrogates both the inflammation and ECM deposition in dermal fibroblasts in vitro [72]. They first identified in the skin and circulation of SSc patients a strong increase of CXCL4, a chemokine that activates monocytes, and M2 macrophages inducing the release of PDGF-BB. CXCL4/PDGF-BB signaling axis in these cells can induce both a pro-inflammatory and a pro-fibrotic phenotype in dermal fibroblasts through the release of IL6 and IL8. Inhibiting the PDGF receptors using crenolanib or siRNA knockdown prevented the inflammatory and fibrogenic phenotype induced by human recombinant PDGF-BB and CXCL4-conditioned macrophage supernatant.

Several second-generation TKI have been developed following the discovery of imatinib. They have been mostly approved for clinical use in patients with chronic myeloid leukemia but have also been evaluated for SSc. In general, second-generation TKI have a broader spectrum of TK targets, encompassing not only PDGFR but also other important downstream signaling pathways [65].

Dasatinib has been evaluated in a small cohort of patients with diffuse SSc in an open-label uncontrolled study, failing to show any significant improvement in skin and lung outcomes [73]. Of note, the patients were treated only for 6 months and then followed up for 18 months. Moreover, an unexpected adverse effect of dasatinib administration was the induction of pulmonary hypertension.

Nilotinib is another second-generation TKI that has been evaluated in a small open-label study enrolling 10 SSc patients, demonstrating not only potential clinical efficacy on cutaneous involvement (although the study was not adequately controlled) but also the ability to induce a significant downregulation of the expression of genes involved in the PDGFR and TGFBR pathways [74]. The onset of QT interval prolongation in 20% of the study participants was a potential adverse effect of nilotinib administration in SSc patients.

Nintedanib is a highly selective TKI targeting PDGF, VEGF, and FGF pathways [75] but, due to its action on other kinases such as Src, MAPK, and ERK, it also inhibits downstream TGF- β pathways [63].

Initially developed in the oncology field, it has subsequently been extensively evaluated for its action in fibrotic diseases [65].

Nintedanib was the first TKI to be approved for the treatment of idiopathic pulmonary fibrosis (IPF) after the INPULSIS trial showed unequivocally that it was effective in halting disease progression as measured by the rate of decline in forced vital capacity (FVC) [76].

Based on preclinical evidence showing that nintedanib was able to inhibit the TGFβ-induced myofibroblast differentiation in preclinical models of SSc [77] and following the success of nintedanib in IPF trials, two large studies evaluating its efficacy on lung involvement in SSc (SSc-ILD) and in other fibrotic lung diseases with a progressive phenotype (PF-ILD) were conducted in recent years.

In the SENSCIS trial that randomized 663 SSc-ILD patients, at 52 weeks the adjusted rate of decline in the FVC was lower using nintedanib instead of placebo, for a between-group difference of 107 mL per year [11]. Of note, despite its efficacy on lung outcomes, nintedanib treatment did not improve skin fibrosis in the SENSCIS trial.

Numerous post-hoc and subgroup analyses of the SENSCIS study suggest that nintedanib efficacy is consistent across the disease spectrum, regardless of predicted %FVC, disease duration, the extent of fibrotic involvement on HRCT, autoantibody status, or SSc [78].

As already mentioned, the subsequent INBUILD study evaluated 663 patients with ILD associated with a variety of systemic diseases, including SSc-ILD [79]. Importantly, patients could be enrolled if they showed features of ILD progression (i.e., a relative decline in the FVC of at least 10% of the predicted value, a relative decline in the FVC of 5% to less than 10% of the predicted value and worsening of respiratory symptoms or an increased extent of fibrosis on high-resolution CT or worsening of respiratory symptoms and an increased extent of fibrosis). The INBUILD study confirmed the remarkable efficacy of nintedanib in slowing the functional decline in patients at high risk of progression, as well as its good safety profile, across all spectra of associated diseases [80].

Following the success of these trials, both the Food and Drug Administration (FDA) and the European Medicines Agency (EMA) approved nintedanib for use in SSc-ILD and PF-ILD.5.

## 9. Conclusions

In this article, we critically reviewed several lines of scientific evidence supporting the notion that PDGF/PDGFR molecular interaction and signaling pathway thereof may play a role in SSc-related fibrosis, affecting multiple organs of the affected individuals. This notion encourages the research of novel therapeutic strategies directed toward this molecular target, which will require aptly designed new animal models for preclinical drug validation and new assays for SSc patient stratification. Indeed, it is entirely possible that upregulation of this signaling pathway is not common to all SSc disease subsets or that this signaling pathway is selectively modulated in distinct disease subsets. Future personalized medicine for SSc patients may benefit from a solution to these issues. 

## Figures and Tables

**Table 1:** Drugs targeting PDGF/PDGFR and their clinical applications. Abbreviations: moAb, monoclonal antibody; IPF, idiopathic pulmonary fibrosis; SSc-ILD, systemic sclerosis-associated interstitial lung disease; PF-ILD, progressive fibrosing interstitial lung disease.

Drug Name	Mechanism of Action	Current Clinical Indications
MOR8457	PDGF-B blocking antibody	Pre-clinical use only
Olaratumab	Recombinant human IgG1 anti-PDGFR moAb	Withdrawn from market
Imatinib	Tyrosine kinase inhibitor	Chronic myeloid leukaemia (CML) Acute lymphoblastic leukaemia (ALL) Myelodysplastic/myeloproliferative diseases Hypereosinophilic syndrome (HES) Gastrointestinal stromal tumours (GIST) Dermatofibrosarcoma protuberans (DFSP)
Crenolanib besylate	Tyrosine kinase inhibitor	Orphan designation for acute myeloid leukaemia
Dasatinib	Tyrosine kinase inhibitor	Chronic myeloid leukaemia (CML) Acute lymphoblastic leukaemia (ALL)
Nilotinib	Tyrosine kinase inhibitor	Chronic myeloid leukaemia (CML)
Nintedanib	Tyrosine kinase inhibitor	IPF SSc-ILD PF-ILD

## Data Availability

Not applicable.

## References

[1] Calderon L.M., Pope J.E. (2021). Scleroderma epidemiology update. Curr. Opin. Rheumatol..

[2] Varga J., Trojanowska M., Kuwana M. (2017). Pathogenesis of systemic sclerosis: Recent insights of molecular and cellular mechanisms and therapeutic opportunities. J. Scleroderma Relat. Disord..

[3] Hoffmann-Vold A.-M., Fretheim H., Meier C., Maurer B. (2020). Circulating biomarkers of systemic sclerosis—Interstitial lung disease. J. Scleroderma Relat. Disord..

[4] Nihtyanova S.I., Denton C.P. (2020). Pathogenesis of systemic sclerosis associated interstitial lung disease. J. Scleroderma Relat. Disord..

[5] Chairta P., Nicolaou P., Christodoulou K. (2017). Genomic and genetic studies of systemic sclerosis: A systematic review. Hum. Immunol..

[6] Dai B., Ding L., Zhao L., Zhu H., Luo H. (2022). Contributions of Immune Cells and Stromal Cells to the Pathogenesis of Systemic Sclerosis: Recent Insights. Front. Pharmacol..

[7] Kuret T., Sodin-Semrl S., Leskosek B., Ferk P. (2021). Single Cell RNA Sequencing in Autoimmune Inflammatory Rheumatic Diseases: Current Applications, Challenges and a Step Toward Precision Medicine. Front. Med..

[8] Szabo I., Muntean L., Crisan T., Rednic V., Sirbe C., Rednic S. (2021). Novel Concepts in Systemic Sclerosis Pathogenesis: Role for miRNAs. Biomedicines.

[9] Blagojevic J., Abignano G., Avouac J., Cometi L., Frerix M., Bellando-Randone S., Guiducci S., Bruni C., Huscher D., Jaeger V.K. (2020). Use of vasoactive/vasodilating drugs for systemic sclerosis (SSc)-related digital ulcers (DUs) in expert tertiary centres: Results from the analysis of the observational real-life DeSScipher study. Clin. Rheumatol..

[10] Kowal-Bielecka O., Fransen J., Avouac J., Becker M., Kulak A., Allanore Y., Distler O., Clements P., Cutolo M., Czirjak L. (2017). Update of EULAR recommendations for the treatment of systemic sclerosis. Ann. Rheum. Dis..

[11] Distler O., Gahlemann M., Maher T.M. (2019). Nintedanib for Systemic Sclerosis-Associated Interstitial Lung Disease. Reply. N. Engl. J. Med..

[12] Andrae J., Gallini R., Betsholtz C. (2008). Role of platelet-derived growth factors in physiology and medicine. Genes. Dev..

[13] Heldin C.H., Westermark B. (1999). Mechanism of action and in vivo role of platelet-derived growth factor. Physiol. Rev..

[14] Yamada K., Hamashima T., Ishii Y., Yamamoto S., Okuno N., Yoshida N., Yamada M., Huang T.T., Shioda N., Tomihara K. (2018). Different PDGF Receptor Dimers Drive Distinct Migration Modes of the Mouse Skin Fibroblast. Cell Physiol. Biochem..

[15] Kazlauskas A. (2017). PDGFs and their receptors. Gene.

[16] Tallquist M., Kazlauskas A. (2004). PDGF signaling in cells and mice. Cytokine Growth Factor Rev..

[17] Klinkhammer B.M., Floege J., Boor P. (2018). PDGF in organ fibrosis. Mol. Aspects Med..

[18] Zhou J., Shao L., Yu J., Huang J., Feng Q. (2021). PDGF-BB promotes vascular smooth muscle cell migration by enhancing Pim-1 expression via inhibiting miR-214. Ann. Transl. Med..

[19] Alvarez R.H., Kantarjian H.M., Cortes J.E. (2006). Biology of platelet-derived growth factor and its involvement in disease. Mayo Clinic Proceedings.

[20] Rogers M.A., Fantauzzo K.A. (2020). The emerging complexity of PDGFRs: Activation, internalization and signal attenuation. Biochem. Soc. Trans..

[21] Valius M., Kazlauskas A. (1993). Phospholipase C-gamma 1 and phosphatidylinositol 3 kinase are the downstream mediators of the PDGF receptor’s mitogenic signal. Cell.

[22] Chiariello M., Marinissen M.J., Gutkind J.S. (2001). Regulation of c-myc expression by PDGF through Rho GTPases. Nat. Cell Biol..

[23] Chen P.H., Chen X., He X. (2013). Platelet-derived growth factors and their receptors: Structural and functional perspectives. Biochim. Biophys. Acta.

[24] Olson L.E., Soriano P. (2009). Increased PDGFRalpha activation disrupts connective tissue development and drives systemic fibrosis. Dev. Cell.

[25] Lei H., Rheaume M.A., Kazlauskas A. (2010). Recent developments in our understanding of how platelet-derived growth factor (PDGF) and its receptors contribute to proliferative vitreoretinopathy. Exp. Eye Res..

[26] Guerit E., Arts F., Dachy G., Boulouadnine B., Demoulin J.B. (2021). PDGF receptor mutations in human diseases. Cell Mol. Life Sci..

[27] Ishii Y., Hamashima T., Yamamoto S., Sasahara M. (2017). Pathogenetic significance and possibility as a therapeutic target of platelet derived growth factor. Pathol. Int..

[28] Medamana J., Clark R.A., Butler J. (2017). Platelet-Derived Growth Factor in Heart Failure. Handb. Exp. Pharmacol..

[29] Rosenbloom J., Macarak E., Piera-Velazquez S., Jimenez S.A. (2017). Human Fibrotic Diseases: Current Challenges in Fibrosis Research. Methods Mol. Biol..

[30] Distler J.H.W., Gyorfi A.H., Ramanujam M., Whitfield M.L., Konigshoff M., Lafyatis R. (2019). Shared and distinct mechanisms of fibrosis. Nat. Rev. Rheumatol..

[31] Roehlen N., Crouchet E., Baumert T.F. (2020). Liver Fibrosis: Mechanistic Concepts and Therapeutic Perspectives. Cells.

[32] White S.J., Chong J.J.H. (2020). Growth factor therapy for cardiac repair: An overview of recent advances and future directions. Biophys. Rev..

[33] Gallini R., Lindblom P., Bondjers C., Betsholtz C., Andrae J. (2016). PDGF-A and PDGF-B induces cardiac fibrosis in transgenic mice. Exp. Cell Res..

[34] Mueller A.A., van Velthoven C.T., Fukumoto K.D., Cheung T.H., Rando T.A. (2016). Intronic polyadenylation of PDGFRalpha in resident stem cells attenuates muscle fibrosis. Nature.

[35] Takamura N., Renaud L., da Silveira W.A., Feghali-Bostwick C. (2021). PDGF Promotes Dermal Fibroblast Activation via a Novel Mechanism Mediated by Signaling Through MCHR1. Front. Immunol..

[36] Clark K.E., Lopez H., Abdi B.A., Guerra S.G., Shiwen X., Khan K., Etomi O., Martin G.R., Abraham D.J., Denton C.P. (2015). Multiplex cytokine analysis of dermal interstitial blister fluid defines local disease mechanisms in systemic sclerosis. Arthritis Res. Ther..

[37] Yamakage A., Kikuchi K., Smith E.A., LeRoy E.C., Trojanowska M. (1992). Selective upregulation of platelet-derived growth factor alpha receptors by transforming growth factor beta in scleroderma fibroblasts. J. Exp. Med..

[38] Ludwicka A., Ohba T., Trojanowska M., Yamakage A., Strange C., Smith E.A., Leroy E.C., Sutherland S., Silver R.M. (1995). Elevated levels of platelet derived growth factor and transforming growth factor-beta 1 in bronchoalveolar lavage fluid from patients with scleroderma. J. Rheumatol..

[39] Liu T., Zhang J., Zhang J., Mu X., Su H., Hu X., Liu W., Zhao E., Li W. (2013). RNA interference against platelet-derived growth factor receptor alpha mRNA inhibits fibroblast transdifferentiation in skin lesions of patients with systemic sclerosis. PLoS ONE.

[40] Tanaka S., Suto A., Ikeda K., Sanayama Y., Nakagomi D., Iwamoto T., Suzuki K., Kambe N., Matsue H., Matsumura R. (2013). Alteration of circulating miRNAs in SSc: miR-30b regulates the expression of PDGF receptor beta. Rheumatology.

[41] Orlando F., Paolini C., Agarbati S., Tonnini C., Grieco A., Capelli C., Introna M., Provinciali M., Gabrielli A., Moroncini G. (2019). Induction of Mouse Lung Injury by Endotracheal Injection of Bleomycin. J. Vis. Exp..

[42] Baroni S.S., Santillo M., Bevilacqua F., Luchetti M., Spadoni T., Mancini M., Fraticelli P., Sambo P., Funaro A., Kazlauskas A. (2006). Stimulatory autoantibodies to the PDGF receptor in systemic sclerosis. N. Engl. J. Med..

[43] Hamaguchi Y. (2010). Autoantibody profiles in systemic sclerosis: Predictive value for clinical evaluation and prognosis. J. Dermatol..

[44] Gabrielli A., Svegliati S., Moroncini G., Avvedimento E.V. (2007). Pathogenic autoantibodies in systemic sclerosis. Curr. Opin. Immunol..

[45] Moroncini G., Mori S., Tonnini C., Gabrielli A. (2013). Role of viral infections in the etiopathogenesis of systemic sclerosis. Clin. Exp. Rheumatol..

[46] Moroncini G., Grieco A., Nacci G., Paolini C., Tonnini C., Pozniak K.N., Cuccioloni M., Mozzicafreddo M., Svegliati S., Angeletti M. (2015). Epitope Specificity Determines Pathogenicity and Detectability of Anti-Platelet-Derived Growth Factor Receptor alpha Autoantibodies in Systemic Sclerosis. Arthritis Rheumatol..

[47] Moroncini G., Cuccioloni M., Mozzicafreddo M., Pozniak K.N., Grieco A., Paolini C., Tonnini C., Spadoni T., Svegliati S., Funaro A. (2017). Characterization of binding and quantification of human autoantibodies to PDGFRalpha using a biosensor-based approach. Anal. Biochem..

[48] Moroncini G., Svegliati Baroni S., Gabrielli A. (2018). Agonistic antibodies in systemic sclerosis. Immunol. Lett..

[49] Svegliati S., Amico D., Spadoni T., Fischetti C., Finke D., Moroncini G., Paolini C., Tonnini C., Grieco A., Rovinelli M. (2017). Agonistic Anti-PDGF Receptor Autoantibodies from Patients with Systemic Sclerosis Impact Human Pulmonary Artery Smooth Muscle Cells Function In Vitro. Front. Immunol..

[50] Luchetti M.M., Moroncini G., Jose Escamez M., Svegliati Baroni S., Spadoni T., Grieco A., Paolini C., Funaro A., Avvedimento E.V., Larcher F. (2016). Induction of Scleroderma Fibrosis in Skin-Humanized Mice by Administration of Anti-Platelet-Derived Growth Factor Receptor Agonistic Autoantibodies. Arthritis Rheumatol..

[51] Kschonsak M., Rouge L., Arthur C.P., Hoangdung H., Patel N., Kim I., Johnson M.C., Kraft E., Rohou A.L., Gill A. (2021). Structures of HCMV Trimer reveal the basis for receptor recognition and cell entry. Cell.

[52] Shim A.H., Liu H., Focia P.J., Chen X., Lin P.C., He X. (2010). Structures of a platelet-derived growth factor/propeptide complex and a platelet-derived growth factor/receptor complex. Proc. Natl. Acad. Sci. USA.

[53] Yuzawa S., Opatowsky Y., Zhang Z., Mandiyan V., Lax I., Schlessinger J. (2007). Structural basis for activation of the receptor tyrosine kinase KIT by stem cell factor. Cell.

[54] Markovic-Mueller S., Stuttfeld E., Asthana M., Weinert T., Bliven S., Goldie K.N., Kisko K., Capitani G., Ballmer-Hofer K. (2017). Structure of the Full-length VEGFR-1 Extracellular Domain in Complex with VEGF-A. Structure.

[55] Oefner C., D’Arcy A., Winkler F.K., Eggimann B., Hosang M. (1992). Crystal structure of human platelet-derived growth factor BB. EMBO J..

[56] Liang L., Yan X.E., Yin Y., Yun C.H. (2016). Structural and biochemical studies of the PDGFRA kinase domain. Biochem. Biophys. Res. Commun..

[57] Papadopoulos N., Lennartsson J. (2018). The PDGF/PDGFR pathway as a drug target. Mol. Aspects Med..

[58] Kuai J., Mosyak L., Brooks J., Cain M., Carven G.J., Ogawa S., Ishino T., Tam M., Lavallie E.R., Yang Z. (2015). Characterization of binding mode of action of a blocking anti-platelet-derived growth factor (PDGF)-B monoclonal antibody, MOR8457, reveals conformational flexibility and avidity needed for PDGF-BB to bind PDGF receptor-beta. Biochemistry.

[59] Musa H., Kaur K., O’Connell R., Klos M., Guerrero-Serna G., Avula U.M., Herron T.J., Kalifa J., Anumonwo J.M., Jalife J. (2013). Inhibition of platelet-derived growth factor-AB signaling prevents electromechanical remodeling of adult atrial myocytes that contact myofibroblasts. Heart Rhythm..

[60] Donovan J., Shiwen X., Norman J., Abraham D. (2013). Platelet-derived growth factor alpha and beta receptors have overlapping functional activities towards fibroblasts. Fibrogenesis Tissue Repair.

[61] Gerber D.E., Gupta P., Dellinger M.T., Toombs J.E., Peyton M., Duignan I., Malaby J., Bailey T., Burns C., Brekken R.A. (2012). Stromal platelet-derived growth factor receptor alpha (PDGFRalpha) provides a therapeutic target independent of tumor cell PDGFRalpha expression in lung cancer xenografts. Mol. Cancer Ther..

[62] Moroncini G., Maccaroni E., Fiordoliva I., Pellei C., Gabrielli A., Berardi R. (2018). Developments in the management of advanced soft-tissue sarcoma—Olaratumab in context. Onco Targets Ther..

[63] Tap W.D., Wagner A.J., Schoffski P., Martin-Broto J., Krarup-Hansen A., Ganjoo K.N., Yen C.C., Abdul Razak A.R., Spira A., Kawai A. (2020). Effect of Doxorubicin Plus Olaratumab vs Doxorubicin Plus Placebo on Survival in Patients With Advanced Soft Tissue Sarcomas: The ANNOUNCE Randomized Clinical Trial. JAMA.

[64] Iqbal N., Iqbal N. (2014). Imatinib: A breakthrough of targeted therapy in cancer. Chemother. Res. Pract..

[65] Mendoza F.A., Piera-Velazquez S., Jimenez S.A. (2021). Tyrosine kinases in the pathogenesis of tissue fibrosis in systemic sclerosis and potential therapeutic role of their inhibition. Transl. Res..

[66] Daniels C.E., Wilkes M.C., Edens M., Kottom T.J., Murphy S.J., Limper A.H., Leof E.B. (2004). Imatinib mesylate inhibits the profibrogenic activity of TGF-beta and prevents bleomycin-mediated lung fibrosis. J. Clin. Investig..

[67] Distler J.H., Jungel A., Huber L.C., Schulze-Horsel U., Zwerina J., Gay R.E., Michel B.A., Hauser T., Schett G., Gay S. (2007). Imatinib mesylate reduces production of extracellular matrix and prevents development of experimental dermal fibrosis. Arthritis Rheum..

[68] Bournia V.K., Evangelou K., Sfikakis P.P. (2013). Therapeutic inhibition of tyrosine kinases in systemic sclerosis: A review of published experience on the first 108 patients treated with imatinib. Seminars in Arthritis and Rheumatism.

[69] Fraticelli P., Gabrielli B., Pomponio G., Valentini G., Bosello S., Riboldi P., Gerosa M., Faggioli P., Giacomelli R., Del Papa N. (2014). Low-dose oral imatinib in the treatment of systemic sclerosis interstitial lung disease unresponsive to cyclophosphamide: A phase II pilot study. Arthritis Res. Ther..

[70] Harrach S., Barz V., Pap T., Pavenstadt H., Schlatter E., Edemir B., Distler J., Ciarimboli G., Bertrand J. (2019). Notch Signaling Activity Determines Uptake and Biological Effect of Imatinib in Systemic Sclerosis Dermal Fibroblasts. J. Investig. Dermatol..

[71] Makino K., Makino T., Stawski L., Mantero J.C., Lafyatis R., Simms R., Trojanowska M. (2017). Blockade of PDGF Receptors by Crenolanib Has Therapeutic Effect in Patient Fibroblasts and in Preclinical Models of Systemic Sclerosis. J. Investig. Dermatol..

[72] van der Kroef M., Carvalheiro T., Rossato M., de Wit F., Cossu M., Chouri E., Wichers C.G.K., Bekker C.P.J., Beretta L., Vazirpanah N. (2020). CXCL4 triggers monocytes and macrophages to produce PDGF-BB, culminating in fibroblast activation: Implications for systemic sclerosis. J. Autoimmun..

[73] Martyanov V., Kim G.J., Hayes W., Du S., Ganguly B.J., Sy O., Lee S.K., Bogatkevich G.S., Schieven G.L., Schiopu E. (2017). Novel lung imaging biomarkers and skin gene expression subsetting in dasatinib treatment of systemic sclerosis-associated interstitial lung disease. PLoS ONE.

[74] Gordon J.K., Martyanov V., Magro C., Wildman H.F., Wood T.A., Huang W.T., Crow M.K., Whitfield M.L., Spiera R.F. (2015). Nilotinib (Tasigna) in the treatment of early diffuse systemic sclerosis: An open-label, pilot clinical trial. Arthritis Res. Ther..

[75] Hilberg F., Roth G.J., Krssak M., Kautschitsch S., Sommergruber W., Tontsch-Grunt U., Garin-Chesa P., Bader G., Zoephel A., Quant J. (2008). BIBF 1120: Triple angiokinase inhibitor with sustained receptor blockade and good antitumor efficacy. Cancer Res..

[76] Richeldi L., du Bois R.M., Raghu G., Azuma A., Brown K.K., Costabel U., Cottin V., Flaherty K.R., Hansell D.M., Inoue Y. (2014). Efficacy and safety of nintedanib in idiopathic pulmonary fibrosis. N. Engl. J. Med..

[77] Huang J., Beyer C., Palumbo-Zerr K., Zhang Y., Ramming A., Distler A., Gelse K., Distler O., Schett G., Wollin L. (2016). Nintedanib inhibits fibroblast activation and ameliorates fibrosis in preclinical models of systemic sclerosis. Ann. Rheum. Dis..

[78] Benfaremo D., Svegliati S., Paolini C., Agarbati S., Moroncini G. (2022). Systemic Sclerosis: From Pathophysiology to Novel Therapeutic Approaches. Biomedicines.

[79] Flaherty K.R., Wells A.U., Cottin V., Devaraj A., Walsh S.L.F., Inoue Y., Richeldi L., Kolb M., Tetzlaff K., Stowasser S. (2019). Nintedanib in Progressive Fibrosing Interstitial Lung Diseases. N. Engl. J. Med..

[80] Wells A.U., Flaherty K.R., Brown K.K., Inoue Y., Devaraj A., Richeldi L., Moua T., Crestani B., Wuyts W.A., Stowasser S. (2020). Nintedanib in patients with progressive fibrosing interstitial lung diseases-subgroup analyses by interstitial lung disease diagnosis in the INBUILD trial: A randomised, double-blind, placebo-controlled, parallel-group trial. Lancet Respir. Med..

